# Genetic Basis of Inherited Retinal Disease in a Molecularly Characterized Cohort of More Than 3000 Families from the United Kingdom

**DOI:** 10.1016/j.ophtha.2020.04.008

**Published:** 2020-10

**Authors:** Nikolas Pontikos, Gavin Arno, Neringa Jurkute, Elena Schiff, Rola Ba-Abbad, Samantha Malka, Ainoa Gimenez, Michalis Georgiou, Genevieve Wright, Monica Armengol, Hannah Knight, Menachem Katz, Mariya Moosajee, Patrick Yu-Wai-Man, Anthony T. Moore, Michel Michaelides, Andrew R. Webster, Omar A. Mahroo

**Affiliations:** 1 UCL Institute of Ophthalmology, University College London, London, United Kingdom; 2 Genetics Service, Moorfields Eye Hospital, London, United Kingdom; 3 North East Thames Regional Genetics Service, Great Ormond Street Institute of Child Health, London, United Kingdom; 4 Cambridge Centre for Brain Repair and MRC Mitochondrial Biology Unit, Department of Clinical Neurosciences, University of Cambridge, Cambridge, United Kingdom; 5 Cambridge Eye Unit, Addenbrooke’s Hospital, Cambridge University Hospitals, Cambridge, United Kingdom; 6 Department of Ophthalmology, University of California, San Francisco, San Francisco, California; 7 Section of Ophthalmology, King’s College London, St. Thomas’ Hospital Campus, London, United Kingdom; 8 Department of Physiology, Development and Neuroscience, University of Cambridge, Cambridge, United Kingdom

**Keywords:** IRD, inherited retinal disease, RP, retinitis pigmentosa

## Abstract

**Purpose:**

In a large cohort of molecularly characterized inherited retinal disease (IRD) families, we investigated proportions with disease attributable to causative variants in each gene.

**Design:**

Retrospective study of electronic patient records.

**Participants:**

Patients and relatives managed in the Genetics Service of Moorfields Eye Hospital in whom a molecular diagnosis had been identified.

**Methods:**

Genetic screening used a combination of single-gene testing, gene panel testing, whole exome sequencing, and more recently, whole genome sequencing. For this study, genes listed in the Retinal Information Network online resource (https://sph.uth.edu/retnet/) were included. Transcript length was extracted for each gene (Ensembl, release 94).

**Main Outcome Measures:**

We calculated proportions of families with IRD attributable to variants in each gene in the entire cohort, a cohort younger than 18 years, and a current cohort (at least 1 patient encounter between January 1, 2017, and August 2, 2019). Additionally, we explored correlation between numbers of families and gene transcript length.

**Results:**

We identified 3195 families with a molecular diagnosis (variants in 135 genes), including 4236 affected individuals. The pediatric cohort comprised 452 individuals from 411 families (66 genes). The current cohort comprised 2614 families (131 genes; 3130 affected individuals). The 20 most frequently implicated genes overall (with prevalence rates per families) were as follows: *ABCA4* (20.8%), *USH2A* (9.1%), *RPGR* (5.1%), *PRPH2* (4.6%), *BEST1* (3.9%), *RS1* (3.5%), *RP1* (3.3%), *RHO* (3.3%), *CHM* (2.7%), *CRB1* (2.1%), *PRPF31* (1.8%), *MY07A* (1.7%), *OPA1* (1.6%), *CNGB3* (1.4%), *RPE65* (1.2%), *EYS* (1.2%), *GUCY2D* (1.2%), *PROM1* (1.2%), *CNGA3* (1.1%), and *RDH12* (1.1%). These accounted for 71.8% of all molecularly diagnosed families. Spearman coefficients for correlation between numbers of families and transcript length were 0.20 (*P* = 0.025) overall and 0.27 (*P* = 0.017), –0.17 (*P* = 0.46), and 0.71 (*P* = 0.047) for genes in which variants exclusively cause recessive, dominant, or X-linked disease, respectively.

**Conclusions:**

Our findings help to quantify the burden of IRD attributable to each gene. More than 70% of families showed pathogenic variants in 1 of 20 genes. Transcript length (relevant to gene delivery strategies) correlated significantly with numbers of affected families (but not for dominant disease).

Monogenic retinal diseases are a major cause of blindness in the pediatric and working-age population in many countries.^1^, ^2^, ^3^ Pathogenic variants in more than 250 genes can give rise to inherited retinal disease (IRD), with multiple modes of inheritance.^4^ For most of these diseases, no medical or surgical treatments exist, but a large number of therapeutic trials are underway.^5^ Currently a commercially available licensed gene-replacement treatment is available for a particular genetic cause: IRD resulting from biallelic variants in *RPE65*.^6^ Because more therapies are likely to become available in the future, with many likely to be specific to a particular genetic cause, it is of increasing relevance to understand the burden of disease attributable to variants in particular genes.

The Genetics Service of Moorfields Eye Hospital oversees the care of the largest number of IRD patients of any one site in the United Kingdom. A significant proportion of these families have a molecular diagnosis, more recently with the advent of parallel nucleotide sequencing and the availability of whole genome sequencing.^7^ When positive genetic diagnoses are made, regarded by the specialist physicians to be in keeping with the patients’ clinical phenotypes and modes of inheritance, these are recorded with the pedigrees in the electronic record. In this study, we interrogated the database to quantify the number of families with pathogenic variants in different genes to build a picture of the most prevalent causes of IRD, within the limitations of such a retrospective analysis. We performed a similar analysis exclusively in patients younger than 18 years to explore the burden of disease in the pediatric cohort. We also investigated relationships between gene transcript length (of relevance when considering development of gene replacement therapies) and number of families affected. Herein, we highlight in particular the 20 most frequently implicated genes, which accounted for more than 70% of the cohort.

## Methods

### Genetic Database Search

Specialist clinics at Moorfields Eye Hospital receive secondary and tertiary referrals for patients with suspected IRD from throughout the United Kingdom. Probands, and in many cases family members, are examined by experienced retinal specialists. After a family is considered solved by the physician (A.R.W., M.M., A.T.M., P.Y.-W.-M., O.M.), the causative gene is recorded within a genetics module within the hospital electronic patient record (OpenEyes Electronic Medical Record, Apperta Foundation, Sunderland, Tyne And Wear, UK). Each pedigree has a unique identifier. In this study, we interrogated the back-end database retrospectively to identify all families with IRD in whom a positive molecular diagnosis had been made. The search date was August 2, 2019, and identified all families in whom a patient encounter had occurred since 2003.

### Genetic Testing Pathway at Moorfields Eye Hospital

Patients are referred to the retinal genetics service when their primary care physician, optometrist or ophthalmologist suspects an IRD. A detailed clinical history is obtained from the patient (and, in the case of children, their parents or guardians), which includes the presence of symptoms, age at onset of symptoms, and order of onset of symptoms, including night vision problems, central vision disturbances, photophobia or hemeralopia, as well as a full medical history and family history (including construction of a pedigree). Patients undergo ophthalmic examination, including visual acuity and intraocular pressure measurement, slit-lamp biomicroscopy, and retinal imaging, comprising spectral-domain OCT and short-wavelength fundus autofluorescence (not always possible in children). Some patients also undergo electroretinography. If patients are suspected by the IRD physician of having an IRD, genetic testing is discussed. In the past, screening was performed most commonly by Sanger sequencing of single genes or small panels. The decision to go ahead with genetic testing in the past was based on a number of factors including the patient’s eagerness to be tested (to help inform prognosis and likelihood of transmission to future generations), the likelihood of positive results, and the possibility of a particular genetic cause that may enable eligibility to treatment trials (early examples were *RPE65* and *CHM*).

In the last decade, next generation sequencing of large gene panels has become more accessible, and testing in our service has been offered more widely and relatively less prone to the above biases. Over the last 5 to 7 years, including the period covering the current cohort of the present study, our service has sought to offer the opportunity for investigating the molecular diagnosis in all patients suspected by the specialist physician of having an IRD. The costs are not borne directly by the patients themselves, but rather are covered by bodies including the National Health Service (NHS) or its research arm, the National Institute of Health Research. Patients with retinitis pigmentosa, other monogenic chorioretinal degenerations, macular dystrophies, cone and cone–rod dystrophies, stationary conditions (including stationary night blindness and achromatopsia), and suspected syndromic retinal dystrophies all undergo genetic testing. Some patients (including those late in life, who may have no children) may decline genetic testing, but most choose to undergo testing. In some cases, including conditions with very mild changes evident on retinal imaging and minimal symptoms, or adult vitelliform maculopathies, where the chances of a positive genetic diagnosis are lower, genetic testing has not been considered uniformly. Figure S1 (available at www.aaojournal.org) broadly illustrates the methods and sequence for genetic testing.

During the past 5 years, the following strategy was adopted for genetic testing. For patients with a retinal dystrophy affecting generalized retinal function (with abnormal full-field scotopic or photopic electroretinography results), a gene panel test was offered covering more than 150 genes known to be implicated in retinal dystrophies (usually performed by the Manchester Centre for Genomic Medicine). In the presence of known autosomal dominant or X-linked inheritance, a restricted panel was requested covering the relevant genes; for X-linked retinal degeneration, this included a request for specific sequencing of the ORF15 exon of *RPGR* because pathogenic variants in this region can be easily missed. For macular dystrophies, restricted panels were requested, frequently using the Stargardt/Macular Dystrophy Panel of the Molecular Vision Laboratory (Hillsboro, OR). Single-gene testing was performed in very few patients in recent years, usually only when a recognizable phenotype implicated a single gene (for example, testing for *RS1* in a male with retinoschisis, a pedigree suggestive of X-linked inheritance, and a negative electroretinogram waveform). Where results of gene panels were negative, but a monogenic disorder was still strongly suspected, further sequencing was initiated if available (as part of either a clinical or research test), including whole genome sequencing.

Whole genome sequencing was available as part of a number of national research projects from 2013 onward. Initially, this was via the National Institute of Health Research Bioresource project (described in a previous publication)^8^ and later as part of the 100 000 Genomes project.^7^ For the latter study, patients were recruited to a pilot study from 2014, with the main study recruiting from 2015 until September 2018. Initial recruitment to the 100 000 Genomes project was for patients who previously showed negative results in initial gene panel screening and for whom DNA samples from additional family members were available. Later, criteria were relaxed, and patients with suspected monogenic disease and no prior testing were eligible, even if samples from family members were not available. The largest number of retinal disease patients was recruited to this study via the retinal genetics service of Moorfields Eye Hospital. When possible, results for patients from our institution are reviewed by a multidisciplinary panel including molecular biologists, clinical geneticists, and the retinal specialist managing the family, and consensus is reached, taking into account prior reports of pathogenicity of the variant,^9^ prevalence in publicly available genome databases, the clinical phenotype, and mode of inheritance, before the molecular diagnosis is established. Approximately 600 probands in the cohort of the present study achieved a molecular diagnosis by whole genome sequencing.

Finally, before access to whole genome sequencing, whole exome sequencing was performed for a number of families (some, but not all, of whom showed negative results previously with single-gene or limited gene panel screening). This testing was performed largely at the Institute of Ophthalmology, University College London, and achieved a molecular diagnosis in approximately 160 families of the cohort reported in this article.

### Inclusion of Genes and Transcript Lengths

For the purposes of the present study, only genes listed on the Retinal Information Network online resource (https://sph.uth.edu/retnet/; accessed October 10, 2019) were included. Transcript lengths for each gene were extracted from online resources (Ensembl, release 94; longest transcript chosen in case of multiple transcripts). We calculated the correlation between numbers of families affected by variants in each gene and the gene’s transcript size. Because the data were not normally distributed, Spearman correlation coefficients were used.

### Consent and Ethical Approval

Patients and relatives gave written informed consent for genetic testing. The study received relevant local research ethics committee approval (Moorfields Eye Hospital and the Northwest London Research Ethics Committee) and conformed to the tenets of the Declaration of Helsinki.

## Results

### Full Cohort

**Figure 1:**
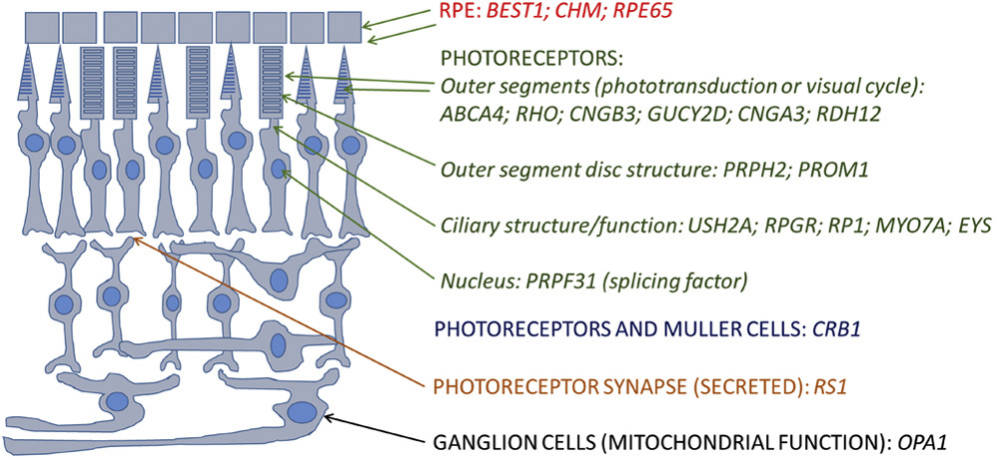
Schematic of the retina showing site of expression of proteins encoded by the 20 most frequently implicated genes in the cohort. RPE = retinal pigment epithelium.

**Figure 2:**
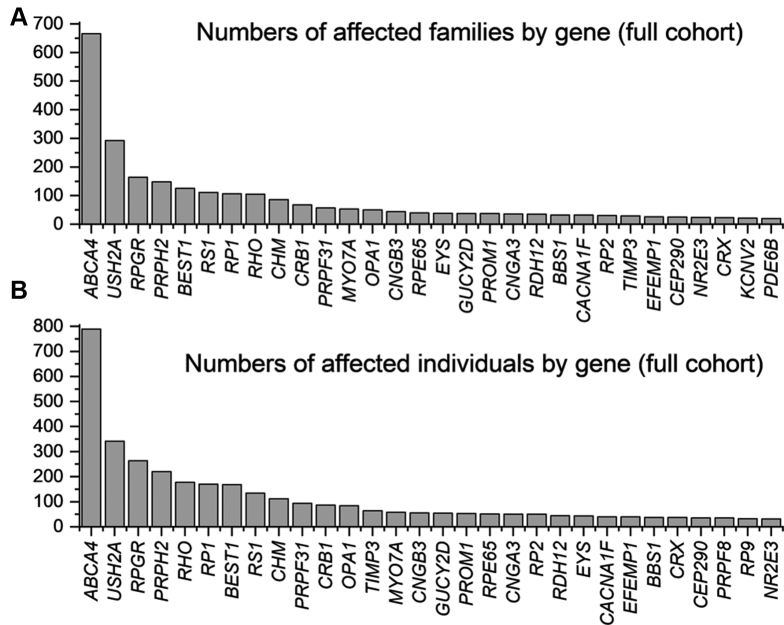
Bar graphs showing the 30 most frequently involved genes in the full cohort. **A**, Genes ranked by numbers of affected families. **B**, Genes ranked by numbers of affected individuals.

Our study identified 4236 individuals from 3195 families with a molecular diagnosis for their disease. Pathogenic variants were found in 135 distinct genes. The full dataset is given in Table S1 (available at www.aaojournal.org). The 20 most frequently implicated genes (by number of affected families) were as follows: *ABCA4* (20.8% of families), *USH2A* (9.1% of families), *RPGR* (5.1% of families), *PRPH2* (4.6% of families), *BEST1* (3.9% of families), *RS1* (3.5% of families), *RP1* (3.3% of families), *RHO* (3.3% of families), *CHM* (2.7% of families), *CRB1* (2.1% of families), *PRPF31* (1.8% of families), *MY07A* (1.7% of families), *OPA1* (1.6% of families), *CNGB3* (1.4% of families), *RPE65* (1.2% of families), *EYS* (1.2% of families), *GUCY2D* (1.2% of families), *PROM1* (1.2% of families), *CNGA3* (1.1% of families), *RDH12* (1.1% of families). These accounted for 71.8% of all molecularly characterized families. Table 1 summarizes key features of these genes, and Figure 1 schematically demonstrates expression by cellular subtype. Figure 2 illustrates numbers affected by the 30 most frequently implicated genes (by number of affected families and numbers of affected individuals, upper and lower panels, respectively). When genes are ranked by numbers of individuals affected, rather than families, autosomal dominant genes, as expected, move upward in rank (e.g., *RHO*, *TIMP3*, *PRPF8*).

**Table 1:** The 20 Most Frequently Implicated Genes in the Full Cohort (by Number of Families)

Gene	Chromosomal Location	No. of Families Affected (%)	Number of Individuals Affected (%)	Methods of Inheritance	Range of Phenotypes in the Literature
*ABCA4*	1p22.1	666 (20.8)	789 (18.6)	Recessive	Stargardt macular dystrophy. cone–rod dystrophy
*USH2A*	1q41	292 (9.1)	342 (8.1)	Recessive	RP, type 2 Usher syndrome
*RPGR*	Xp11.4	164 (5.1)	263 (6.2)	X-linked	RP, cone or cone–rod dystrophy
*PRPH2*	6p21.1	148 (4.6)	220 (5.2)	Dominant and recessive	Pattern dystrophy, RP
*BEST1*	11q12.3	125 (3.9)	168 (4.0)	Dominant and recessive	Best disease, autosomal recessive bestrophinopathy
*RS1*	Xp22.13	111 (3.5)	134 (3.2)	X-linked	X-linked retinoschisis
*RP1*	8q12.1	106 (3.3)	170 (4.0)	Dominant and recessive	RP
*RHO*	3q22.1	105 (3.3)	177 (4.2)	Dominant and recessive	RP, stationary night blindness
*CHM*	Xq21.2	86 (2.7)	112 (2.6)	X-linked	Choroideremia
*CRB1*	1q31.3	68 (2.1)	86 (2.0)	Recessive	LCA, RP, macular dystrophy
*PRPF31*	19q13.42	57 (1.8)	94 (2.2)	Dominant	RP
*MYO7A*	11q13.5	53 (1.7)	58 (1.4)	Recessive	Type 1 Usher syndrome
*OPA1*	3q29	50 (1.6)	84 (2.0)	Dominant	Optic atrophy, optic atrophy with sensorineural hearing loss
*CNGB3*	8q21.3	44 (1.4)	55 (1.3)	Recessive	Achromatopsia, cone dystrophy
*RPE65*	1p31.2	39 (1.2)	51 (1.2)	Recessive and dominant	LCA, RP
*EYS*	6q12	38 (1.2)	43 (1.0)	Recessive	RP
*GUCY2D*	17p13.1	37 (1.2)	54 (1.3)	Recessive and dominant	LCA, RP, cone or cone–rod dystrophy
*PROM1*	4p15.32	37 (1.2)	53 (1.2)	Recessive and dominant	Macular dystrophy, cone–rod dystrophy, RP
*CNGA3*	2q11.2	36 (1.1)	50 (1.2)	Recessive	Achromatopsia, cone dystrophy
*RDH12*	14q24.1	35 (1.1)	44 (1.0)	Recessive and dominant	LCA, RP

LCA = Leber congenital amaurosis; RP = retinitis pigmentosa.

Methods of inheritance and range of possible phenotypes are given.

Among the families, 85.3% showed causative variants in autosomal genes (most frequently *ABCA4*, *USH2A*, *PRPH2*, and *BEST1*), 13.7% in X-linked genes (most commonly *RPGR*, *RS1*, and *CHM*), and 1.0% in mitochondrial genes (including those implicated in Leber hereditary optic neuropathy and maternally inherited diabetes and deafness). Of the autosomal genes, most were genes in which variants acted exclusively recessively (52.6% of all families); 8.2% of families showed variants in genes in which disease-causing variants are solely dominant, and 24.5% of families showed variants in genes that can contain dominant or recessively acting pathogenic variants.

For patients with autosomal dominant retinitis pigmentosa (RP), the most frequently associated genes were *RHO*, *RP1*, and *PRPF31*; for X-linked and autosomal recessive forms of RP, the most frequently associated genes were *RPGR* and *USH2A*, respectively. For macular dystrophies, the most common gene by far was *ABCA4* (autosomal recessive), whereas *PRPH2* and *BEST1* were implicated frequently in autosomal dominantly inherited macular dystrophies.

**Figure 3:**
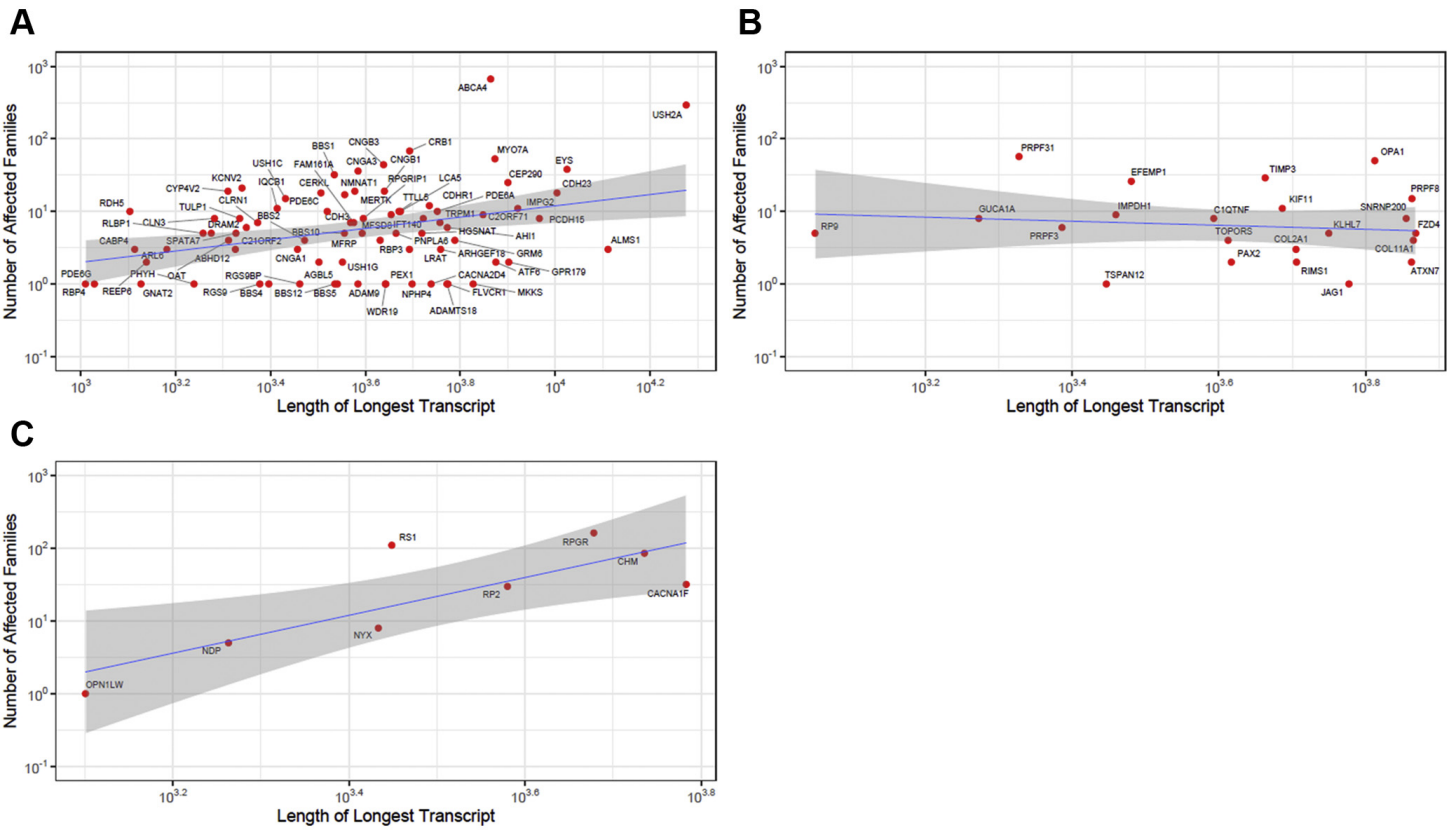
Graphs showing the numbers of affected families plotted against transcript length. **A**, Autosomal genes in which pathogenic variants act exclusively recessively (Spearman correlation coefficient, 0.27; *P* = 0.017). **B**, Autosomal genes in which pathogenic variants act exclusively dominantly (Spearman correlation coefficient, –0.17; *P* = 0.459). **C**, X-linked genes (Spearman correlation coefficient, 0.71; *P* = 0.047).

For all genes (excluding mitochondrial), the Spearman coefficient of correlation between number of families and transcript size was 0.20 (*P* = 0.025). Figure 3 separately plots numbers of families against transcript size for autosomal genes in which variants act solely recessively (Fig 3A), autosomal genes in which variants are solely dominant (Fig 3B), and X-linked genes (Fig 3C). A significant positive correlation was observed in each case, with the exception of autosomal dominant genes.

### Ethnicity and Phenotypic Subtypes

Data on ethnicity were not recorded uniformly for all of the patients in the genetics service. However, these data were available almost completely for all of the families recruited from our service for whole genome sequencing via the 100 000 Genomes project. This distribution of ethnicity is representative of our cohort. Of 1287 IRD probands recruited to the main project, 62.2% were white, 17.9% were Asian (largely South Asian), 7.0% were black (African, Caribbean, and other black background), and 1.8% were of mixed race or ethnicity. These reflect a combination of the demographics of London and the wider United Kingdom (given that many patients seen in the genetics service are referred from outside London, and sometimes from outside England). The full ethnic distribution is presented in Figure S2 (available at www.aaojournal.org).

We also were not able to extract phenotypic subgroups readily in an automated way from the electronic data record because of the variability in data entry and diagnostic labelling. However, this information was available for the above 1287 probands. Of this group, the largest diagnostic category was rod–cone dystrophy (49%), followed by macular dystrophy (35%), inherited optic neuropathy (4.9%), Leber congenital amaurosis or early-onset severe retinal dystrophy (3%), rod dysfunction syndrome (3%), cone dysfunction syndrome (2%), and familial exudative vitreoretinopathy (2%). Entries were limited to these categories; patients with cone dystrophies were entered within the macular dystrophy or cone dysfunction categories. The proportions were similar within each of the major ethnic categories (white, Asian, black), and pairwise comparisons did not reveal significant differences, except for a smaller proportion of Asian patients (29.4%) being diagnosed with macular dystrophy than the corresponding proportions of white (36.6%) or black (43.3%) patients and more Asian patients being diagnosed with familial exudative vitreoretinopathy (4.3%) than black patients (0%). However, the differences were no longer significant after correction for multiple testing.

### Pediatric Cohort

**Figure 4:**
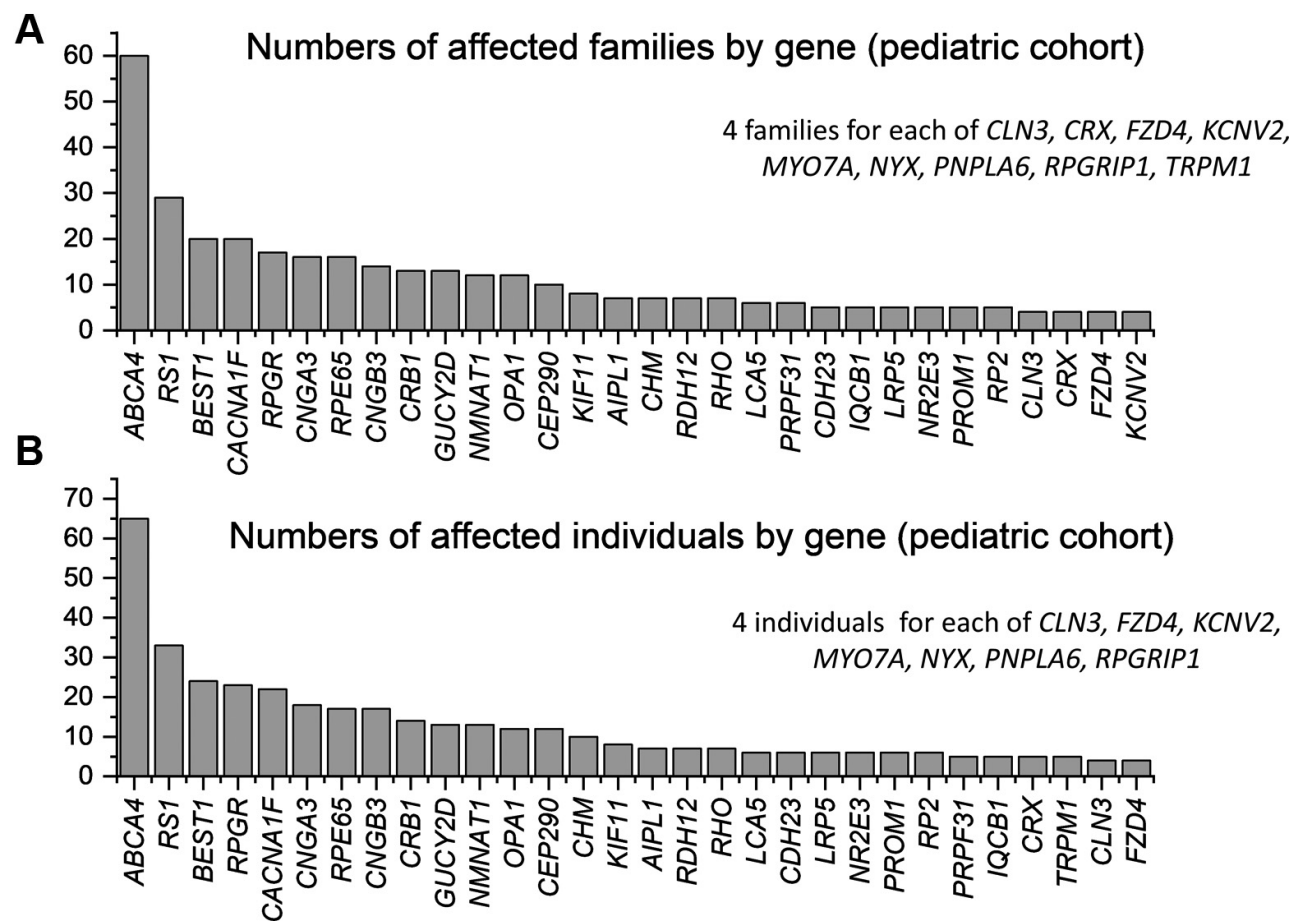
Bar graphs showing the 30 most frequently involved genes in the cohort younger than 18 years. **A**, Genes ranked by numbers of affected families. **B**, Genes ranked by numbers of affected individuals.

To explore burden of disease in a pediatric population, an additional analysis was performed separately for patients younger than 18 years. Our search yielded 452 individuals from 411 molecularly diagnosed families with variants in 66 genes. This dataset is given in Table S2 (available at www.aaojournal.org). The 69 genes implicated in the overall dataset that were not present in those younger than 18 years in our cohort are listed separately in Table S3 (available at www.aaojournal.org). Figure 4 illustrates the 30 most frequently encountered genes by number of affected families or affected individuals (for comparison with Fig 2). In this cohort, the top 20 genes accounted for 73% of the cohort (by number of affected families).

In the pediatric cohort, 78.8% of families showed causative variants in autosomal genes (most frequently *ABCA4* and *BEST1*), 20.7% in X-linked genes (most commonly *RS1* and *RPGR*), and 0.5% in mitochondrial genes (associated with Leber hereditary optic neuropathy). Of the autosomal genes, most were genes in which variants acted exclusively recessively (47.7% of all families); 8.8% of families showed variants in genes in which disease-causing variants are solely dominant; 22.4% showed variants in genes that can contain dominant or recessively acting pathogenic variants. In comparison with the overall cohort, the proportion of families with causative variants in X-linked genes was significantly greater in the pediatric cohort (*P* < 0.001).

The X-linked genes in both cohorts included *RPGR* (associated with RP or cone–rod dystrophy), *RS1* (associated with X-linked retinoschisis), *CHM* (choroideremia), *CACNA1F* (incomplete congenital stationary night blindness), *RP2* (associated with RP), *NYX* (complete congenital stationary night blindness), and *NDP* (associated with Norrie disease or X-linked familial exudative vitreoretinopathy). Of these, some affected female patients were seen in the overall cohort with disease associated with *RPGR*, *CHM*, and *RP2*, consistent with the possibility of female patients demonstrating symptoms. These tend to be milder and usually appear later in life than in male patients. Thus, in the pediatric cohort, very few affected female patients were seen for the X-linked genes (only 2 female patients were recorded as being affected by variants in *RPGR*).

Notably also, *PRPH2* and *USH2A* were not among the most frequently implicated genes, in contrast to the overall cohort, consistent with variants in these genes more frequently leading to visual impairment later in life, relative to some of the other commonly associated genes. However, some genes associated with congenital stable, or very early-onset progressive, visual impairment were among the top 10 genes in the pediatric cohort, but not in the overall cohort, as follows: *CACNA1F*, associated with incomplete congenital stationary night blindness; *CNGA3* and *CNGB3*, associated with achromatopsia; and *RPE65* and *CRB1*, associated with Leber congenital amaurosis or early-onset severe retinal dystrophy.

### Current Cohort

To reduce the bias inherent in the inclusion of all molecularly characterized families, some of whom will not have accessed the clinical services for many years, but whose data appears because of a historic and specific interest in their disorder or ease of genetic testing for a specific gene, we conducted a third data search. This was limited to families in which patients had undergone an encounter with our service within the last 2 to 3 years (specifically between January 1, 2017, and the search date, August 2, 2019). This may include both clinical examination or a virtual clinic consisting of correspondence with patients informing them of their genetic results if these have only recently come to light.

**Figure 5:**
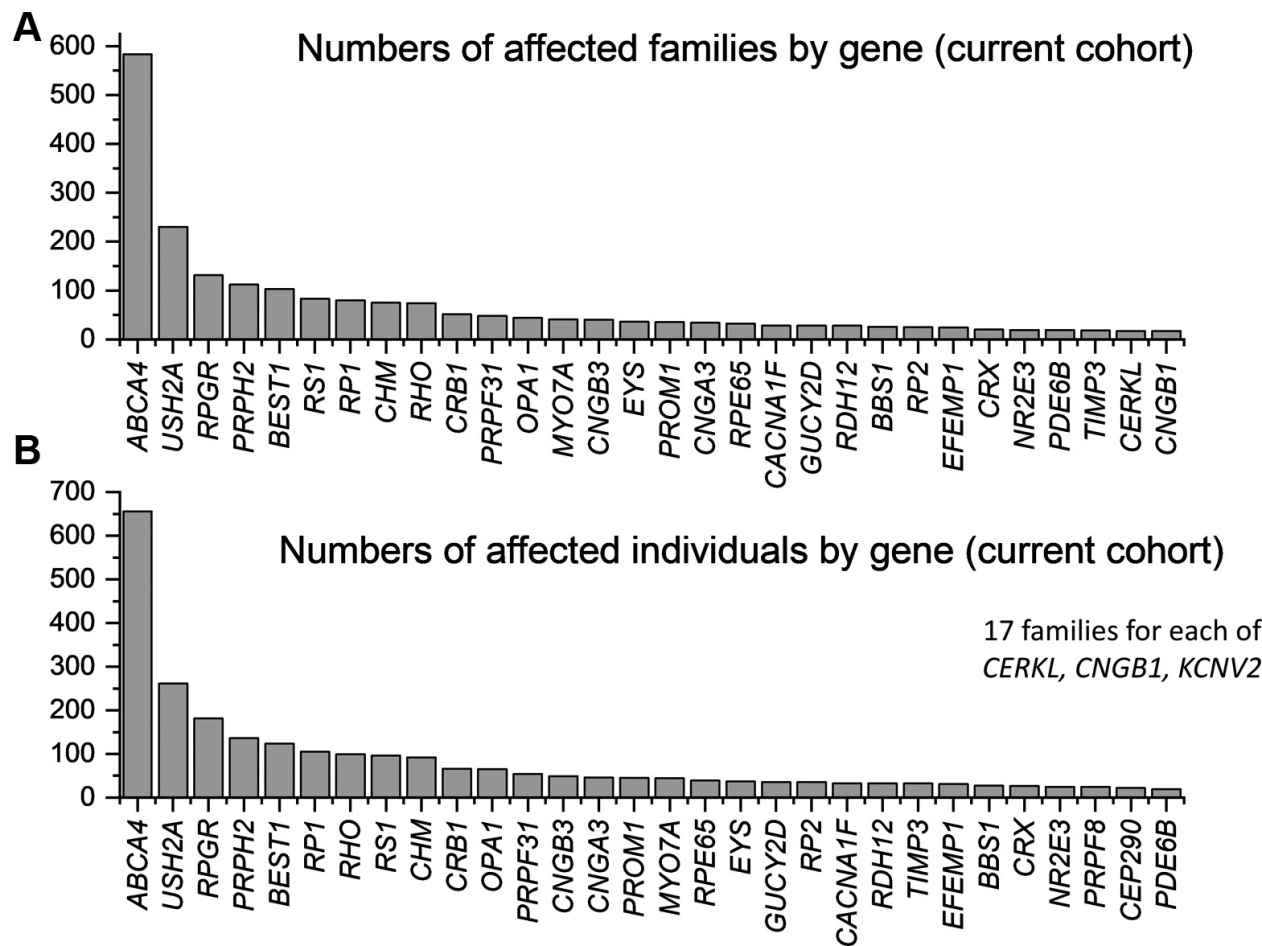
Bar graphs showing the 30 most frequently involved genes in the current cohort (in which a patient encounter had occurred within the preceding 2.5 years). **A**, Genes ranked by numbers of affected families. **B**, Genes ranked by numbers of affected individuals.

This current cohort yielded 3130 individuals from 2614 distinct, molecularly characterized families. Causative variants were in 131 genes. The full dataset is given in Table S4 (available at www.aaojournal.org). The 20 most frequently implicated genes accounted for 71.2% of the total number of families. Figure 5 illustrates the 30 most common genes by number of families affected and by numbers of individuals, in the same format as Figure 2, Figure 4. The order of genes was very similar to that in the overall cohort. The proportions of families with causative variants in X-linked genes (13.5%), in autosomal genes in which pathogenic variants act exclusively dominantly (8.3%), in autosomal genes in which variants act exclusively recessively (53.7%), in autosomal genes in which pathogenic variants can be dominant or recessive (23.5%), and in mitochondrial genes (0.9%) were not significantly different from the corresponding proportions in the overall cohort.

## Discussion

In this study, we investigated the burden of IRD attributable to different genes in a large United Kingdom cohort of 3197 molecularly diagnosed families (more than 4000 affected individuals). This is the largest published molecularly solved IRD cohort to date, as far as the authors are aware. Our families showed variants in 135 genes that are associated with IRD on the Retinal Information Network online resource. We found that the 20 most frequently involved genes accounted for more than 70% of the cohort. Of these 20 genes, 1 gene (*RPE65*) is the subject of licensed commercially available gene therapy, and a further 7 genes (*ABCA4*, *CHM*, *CNGA3*, *CNGB3*, *MY07A*, *RPGR*, and *RS1*) are subjects of experimental gene-replacement trials.^5^

The most frequently encountered gene was *ABCA4* (causing Stargardt macular dystrophy or cone–rod dystrophy). The most frequent gene accounting for autosomal recessive RP was *USH2A*. For autosomal dominant RP, the most commonly encountered genes were *RHO*, *RP1*, and *PRPF31*. A significant proportion (nearly 40%) of X-linked retinopathy was the result of variants in *RPGR*. Although, as expected, the vast majority of affected individuals with pathogenic variants in X-linked genes were male, some affected female patients were recorded in the *RPGR*, *CHM*, and *RP2* gene groups, consistent with the known possibility of female patients being affected; no affected female patients were found to be associated with the other X-linked genes in the overall cohort (and no affected *CHM* or *RP2* female patients were found in the pediatric cohort).

We additionally analyzed genes implicated in our pediatric cohort and a more current subsection of the full cohort in an attempt to mitigate partially the effect of historical bias in the overall cohort. Although the current cohort was very similar to the overall cohort, some important differences were noted in the pediatric cohort. The proportion of families affected by variants in X-linked genes was significantly higher in the pediatric cohort. This may reflect the earlier onset and severity of some of the X-linked diseases, and also the likelihood of earlier diagnosis in individuals in whom parents and clinicians are alerted by a positive family history (which is often absent in autosomal recessive conditions, these forming the largest proportion of both cohorts). A number of genes noted in the overall cohort were absent in the pediatric cohort, which may reflect rarity of these variants (and hence their absence in a cohort of smaller size) or that many genotypes lead to later-onset visual impairment. *PRPH2* and *USH2A* were among the 5 most frequently implicated genes in the overall cohort, but not the pediatric cohort, consistent with older ages of diagnosis (or significant visual impairment) in many cases. Conversely, a number of genes with congenital or early-onset visual impairment appeared more frequently in the pediatric cohort (detailed in “Results”).

### Findings in Other Cohorts

A number of prior studies have examined IRD cohorts.^4^^,^^8^^,^^10^, ^11^, ^12^, ^13^, ^14^, ^15^, ^16^, ^17^, ^18^, ^19^, ^20^, ^21^, ^22^, ^23^, ^24^, ^25^, ^26^ Table 2 presents the most frequently involved genes in many of these published studies over the last 3 to 4 years. Obvious similarities exist in terms of genes affected across diverse geographic regions. However, interesting differences exist, too. Variants in *FAM161A* account for a substantial proportion of disease in a large Israeli cohort.^11^ Variants in *EYS* were a more frequent cause of disease than were *USH2A* variants in Korean patients^12^; this also has been reported in a large cohort of Japanese RP patients.^26^ In a large Chinese RP cohort, *CYP4V2* was the second most implicated gene after *USH2A*.^27^ Differences between populations can reflect founder effects and are important in guiding genetic testing and future interpretation of results of whole genome sequencing. In addition, a relative paucity of studies of IRD cohorts in other large regions, such as Africa, is apparent and worthy of addressing in future investigations.

**Table 2:** Selected Previous Studies in Inherited Retinal Disease Cohorts

Study Author(s) by Year	Study Cohort or Country	No. of Molecularly Diagnosed (No. of Genes)	Most Frequently Implicated Genes
Current study	United Kingdom	4241 individuals from 3197 families (135 genes)	By family: *ABCA4*, *USH2A*, *RPGR*, *PRPH2*, *BEST1*; by individual: *ABCA4*, *USH2A*, *RPGR*, *PRPH2*, *RHO*
2019			
Khan^10^	United Arab Emirates (children)	71 individuals (26 genes)	*ABCA4*, *KCNV2*, *CRB1*, *CNGA3*
Sharon et al^11^	Israel	1369 families (129 genes)	*ABCA4*, *USH2A*, *FAM161A*, *CNGA3*, *EYS*
Holtan et al^12^	Norway	207 patients (56 genes)	*ABCA4*, *USH2A*, *BEST1*, *RHO*, *RS1*
Avela et al^13^	Finland (children)	41 families (17 genes)	*RS1*, *GUCY2D*, *RPGR*
Kim et al^14^	Korea	38 individuals (24 genes)	*ABCA4*, *EYS*, *PDE6B*, *USH2A*, *PDE6A*, *GUCY2D*
Tayebi et al^15^	Iran	36 families (19 genes)	*ABCA4*, *RPE65*, *CERKL*, *RPGRIP1*
2018			
Motta et al^16^	Brazil	400 individuals (66 genes)	*ABCA4*, *CEP290*, *USH2A*, *CRB1*, *RPGR*
Wang et al^17^	China	132 families (47 genes)	*USH2A*, *RPGR*, *CYP4V2*, *ABCA4*, *CRB1*, *RHO*
2017			
Stone et al^18^	United States	760 families (104 genes)	*ABCA4*, *USH2A*, *RPGR*, *RHO*, *PRPH2*
Carss et al^8^	United Kingdom	404 individuals (94 genes)	*ABCA4*, *USH2A*, *EYS*, *RP1*, *CACNA1F*, *RPGR*
Dockery et al^19^∗	Ireland (adults)	357 families (59 genes)	*ABCA4*, *USH2A*, *BBS1*, *RHO*, *RP1*
Ellingford et al^20^	UK, genomic laboratory	271 individuals (62 genes)	*USH2A*, *CRB1*, *ABCA4*, *CERKL*, *CEP290*
Haer-Wigman et al^21^	Netherlands	136 individuals (56 genes)	*USH2A*, *EYS*, *ABCA4*, *RPGR*, *GUCY2D*, *PDE6B*
Riera et al^22^	Spain	42 individuals (29 genes)	*ABCA4*, *USH2A*, *PDE6A*, *CRB1*, *EYS*, *GUCY2D*, *PDE6B*
2016			
Tiwari et al^23^	Switzerland	58 individuals (18 genes)	*ABCA4*, *C2orf71*, *RP1*, *CEP290*, *FLVCR1*, *CRB1*
Bernardis et al^24^	Italy	52 individuals (16 genes)	*ABCA4*, *USH2A*, *RPGR*, *CNGB1*, *BEST1*

Some authors report results from gene panel or whole exome or whole genome testing, leading to likely underrepresentation of disorders diagnosed with single-gene testing. Studies restricted to specific phenotypes (e.g., retinitis pigmentosa) are not shown. Some smaller cohorts are included to allow wider geographical representation. The right-hand column gives the most frequently implicated genes. (In most cases, these are the top 5, but where multiple genes contributed the same proportion, additional genes may be included.) For some 2019 studies, year published relates to year of online publication (print publication in some cases was in 2020).

∗ Some data relating to this study were taken from the publication Farrar et al.^4^

Rates of consanguinity also differ between population groups. When consanguinity or endogamy is more common, autosomal recessive diseases associated with homozygous variants will be more likely. Recently published findings from the United Arab Emirates^10^ showed that the most frequently implicated genes in a pediatric cohort were those in which pathogenic variants are inherited recessively, with many associated with homozygous variants. In contrast, in our pediatric cohort, after *ABCA4*, the next 4 most frequently inherited genes were associated with X-linked or predominantly autosomal dominant disease (although recessive disease did feature in a number of the top 20 genes). Eliciting a history of consanguinity can be helpful not just in selecting genes for screening, but also in interpreting results of whole genome sequencing, where preliminary focus concentrates on regions of homozygosity. Other methods of inheritance of course are possible, even in consanguineous cohorts. For example, in the pediatric study from the United Arab Emirates, *RS1* (X-linked) and *BEST1* (usually associated with autosomal dominant disease) also featured in a number of families.^10^

Genetic testing strategies and their accessibility also differ among countries: those in which targeted restricted gene panels are used selectively in patients with recognizable phenotypes could lead to a greater reported prevalence of those genes (for example, possibly contributing to the higher prevalence of *KCNV2* retinopathy, which has a pathognomonic electroretinography phenotype,^28^ in the United Arab Emirates study).^10^ The availability of whole genome sequencing to a proportion of our cohort, as part of a national research project, and access to particular gene panels with testing paid for by the National Health Service or its research arm may not be applicable to other countries with different accessibility to clinical and research tests and different arrangements for reimbursement.

### Correlations with Transcript Length

The length of the transcript is of relevance in the context of gene replacement therapy; a limit exists to the size of cDNA that can be delivered by different virus vectors. Adenoassociated viruses have been a vector of choice for ocular gene therapy trials, targeting retinal cells with relatively low immunogenicity, but their capacity is limited.^5^ We explored transcript lengths and relationships with numbers of families affected. We found a weak, but statistically significant, correlation in the overall cohort. For autosomal genes in which pathogenic variants act recessively, the correlation remained significant, whereas no apparent correlation was found for dominant genes.

Longer transcripts may be expected, by virtue of their length, to contain more sites in which a variant potentially can bring about premature termination or loss of function, which is the usual method of action in recessive disease. Thus, a greater prevalence of pathogenic variants in longer genes might be anticipated. For many dominant diseases, however, loss of function variants in many cases do not cause disease. Pathogenicity frequently is consequent on a gain of function or specific effects of mutations (for some genes, only a few dominantly acting variants have been identified), and so prevalence of disease may not be expected to correlate in the same way with transcript length. In contrast, X-linked disease often is a result of loss of the single functioning allele in male patients (and again longer genes may have more sites at which mutation can lead to premature termination or loss of function); this may explain the significant correlation between number of affected families and transcript size observed for X-linked genes (although the number of genes here is relatively small).

### Study Limitations

Our findings should be taken in the context of a number of important limitations inherent in such a retrospective study. The study relies on prior data entry, which may be incomplete or inconsistent or in some cases contain errors, although efforts are made to correct these when they come to light. It is likely that a number of genes are overrepresented, including those discovered earlier, those more amenable to sequencing by earlier methods, or those in which historic or current interest exists particularly in light of potential gene-specific therapies. This effect will lessen over time, as more patients have undergone whole genome sequencing, permitting unbiased analysis of data. Detection of structural variants and variants in noncoding regions still can be challenging, as can detection of pathogenic variants in the repetitive ORF15 exon of *RPGR*. The latter can be easily missed in whole genome sequencing, and so the burden of disease resulting from *RPGR* may be underestimated. In addition, some of the earlier results predate current guidelines^9^ and the availability of large databases of common variants, and so variants previously classified as pathogenic may no longer be regarded as such.

We sought to mitigate partly the effect of historical biases by performing a time-limited analysis of more current patient data, which represented a large proportion of the overall cohort. During the period pertaining to the current cohort, clinical or research genetic testing was offered routinely to all patients who were reviewed in clinic and were suspected by the specialist physician of having an IRD, with no direct cost borne by the patient.

A further source of potential ascertainment bias relates to the types of patients managed in our service. Although Moorfields Eye Hospital cares for both children and adults, some of the more severe syndromic conditions tend to be managed in other specialist centers, with multidisciplinary medical input. Thus, these disorders are likely to be underrepresented in our cohort.

Given the retrospective nature of the study, we were unable to ascertain a number of other potentially useful data. The total number of patients enrolled for genetic testing was not available, thus precluding calculation of a molecularly solved rate for the entire cohort. Whole genome sequencing, when available, was offered initially to patients who had shown negative results with prior gene panels, but was offered later to all patients, thus making this a mixed group. In a prior study partly from our service,^8^ 63% of patients with no prior testing achieved a molecular diagnosis from whole exome or whole genome sequencing, compared with 54% of those who previously had shown negative results on prior gene panels.

Also, the date of first symptoms or first clinical diagnosis was not available. Because of variability in data entry, we could not extract readily the proportions attributable to particular variants of each gene, race or ethnicity data by genotype, or the specific frequency of phenotypic subgroups for the entire cohort, but these are useful subjects for further exploration. Our findings thus give a sense of relative burdens of disease attributable to different genes in a large multiethnic United Kingdom-based cohort but may not apply precisely to other populations with different ethnic compositions (as discussed in relation to Table 2) or with different availabilities or strategies for genetic testing.

With parallel developments in genomic testing and novel therapies, we envision that it will become a standard of care to seek the molecular diagnosis in most IRD patients. Quantification of disease burden attributable to particular genes, and particular genetic variants, in diverse populations will be important in both guiding individual patient management and planning within healthcare systems to address this important cause of blindness.
