# Supplementary material for: Genetic Basis of Inherited Retinal Disease in a Molecularly Characterized Cohort of More Than 3000 Families from the United Kingdom
Source: Ophthalmology. 2020 Oct;127(10):1384–94. doi: 10.1016/j.ophtha.2020.04.008 (PMC7520514; doi:10.1016/j.ophtha.2020.04.008)
Supplement: Figure S1 [file mmc1.pdf]

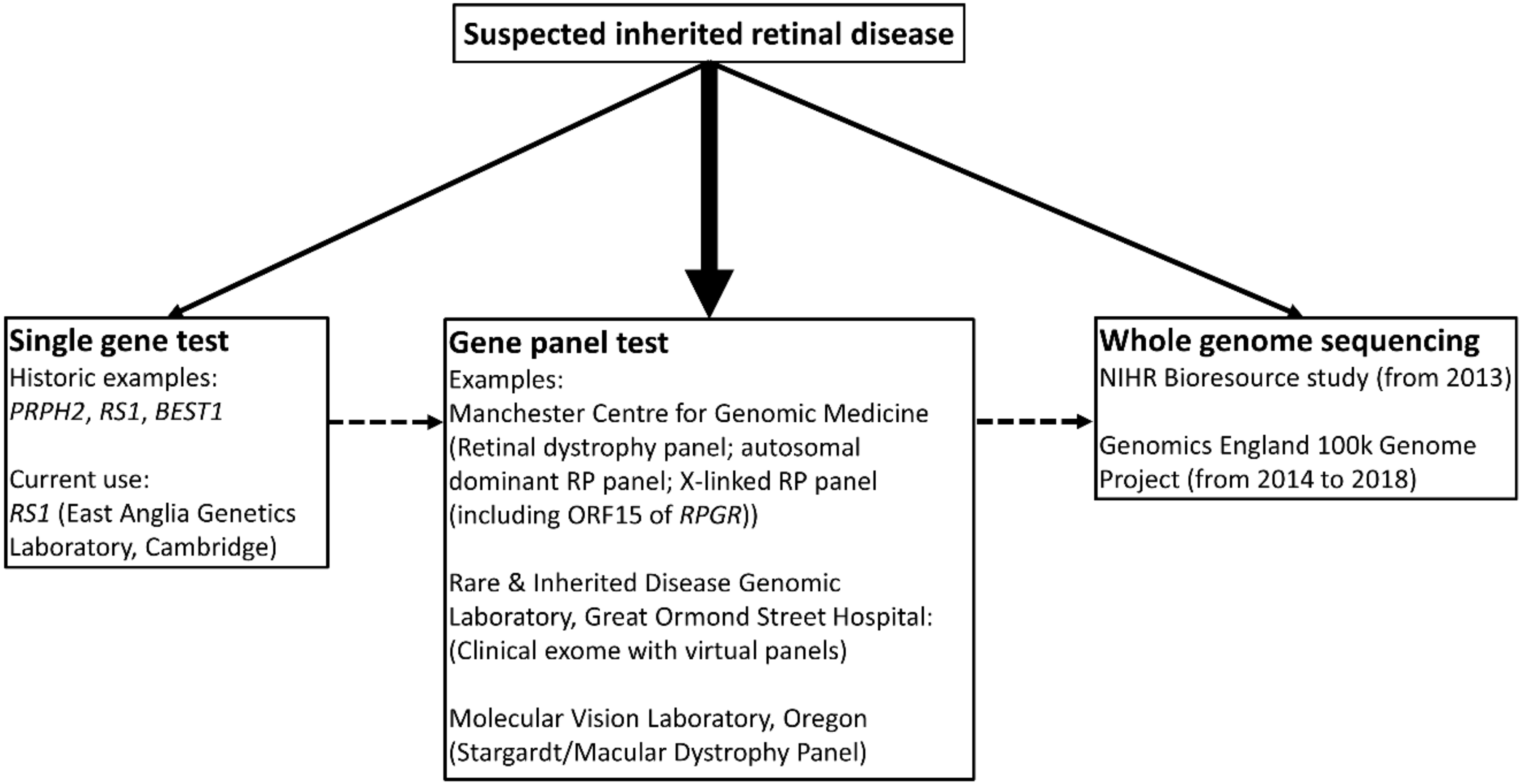

Suspected inherited retinal disease
Single gene test
Historic examples:
PRPH2, *RS1*, *BEST1*
Current use:
RS1 (East Anglia Genetics Laboratory, Cambridge)
Gene panel test
Examples:
Manchester Centre for Genomic Medicine (Retinal dystrophy panel; autosomal dominant RP panel; X-linked RP panel (including ORF15 of *RPGR*))
Rare & Inherited Disease Genomic Laboratory, Great Ormond Street Hospital: (Clinical exome with virtual panels)
Molecular Vision Laboratory, Oregon (Stargardt/Macular Dystrophy Panel)
Whole genome sequencing
NIHR Bioresource study (from 2013)
Genomics England 100k Genome Project (from 2014 to 2018)

Supplementary Figure 1. Strategy for genetic testing in our cohort. Testing was performed via a combination of single gene tests, gene panels and whole genome sequencing. The majority of patients in this molecularly characterised cohort achieved a diagnosis via gene panel testing (middle box, associated with large solid arrow). Historically, single-gene tests were used (left box). If patients were negative for single gene testing, gene panel testing was employed (left hand dashed arrow). More recently, for a period of 5 years (2013 to 2018) patients were recruited for whole genome sequencing (initially whole exome, then whole genome sequencing, for the NIHR Bioresource study), represented by the right-hand box. Some of these patients had previously tested negative for single gene or gene panel tests (indicated by horizontal dashed arrows) and some were recruited directly (right solid arrow). For a limited period prior to this, whole exome sequencing (not shown) was performed as part of research studies (largely based at the Institute of Ophthalmology, University College London), which led the molecular diagnosis in 162 families.
