# Supplementary material for: Genetic Basis of Inherited Retinal Disease in a Molecularly Characterized Cohort of More Than 3000 Families from the United Kingdom
Source: Ophthalmology. 2020 Oct;127(10):1384–94. doi: 10.1016/j.ophtha.2020.04.008 (PMC7520514; doi:10.1016/j.ophtha.2020.04.008)
Supplement: Figure S2 [file mmc2.pdf]

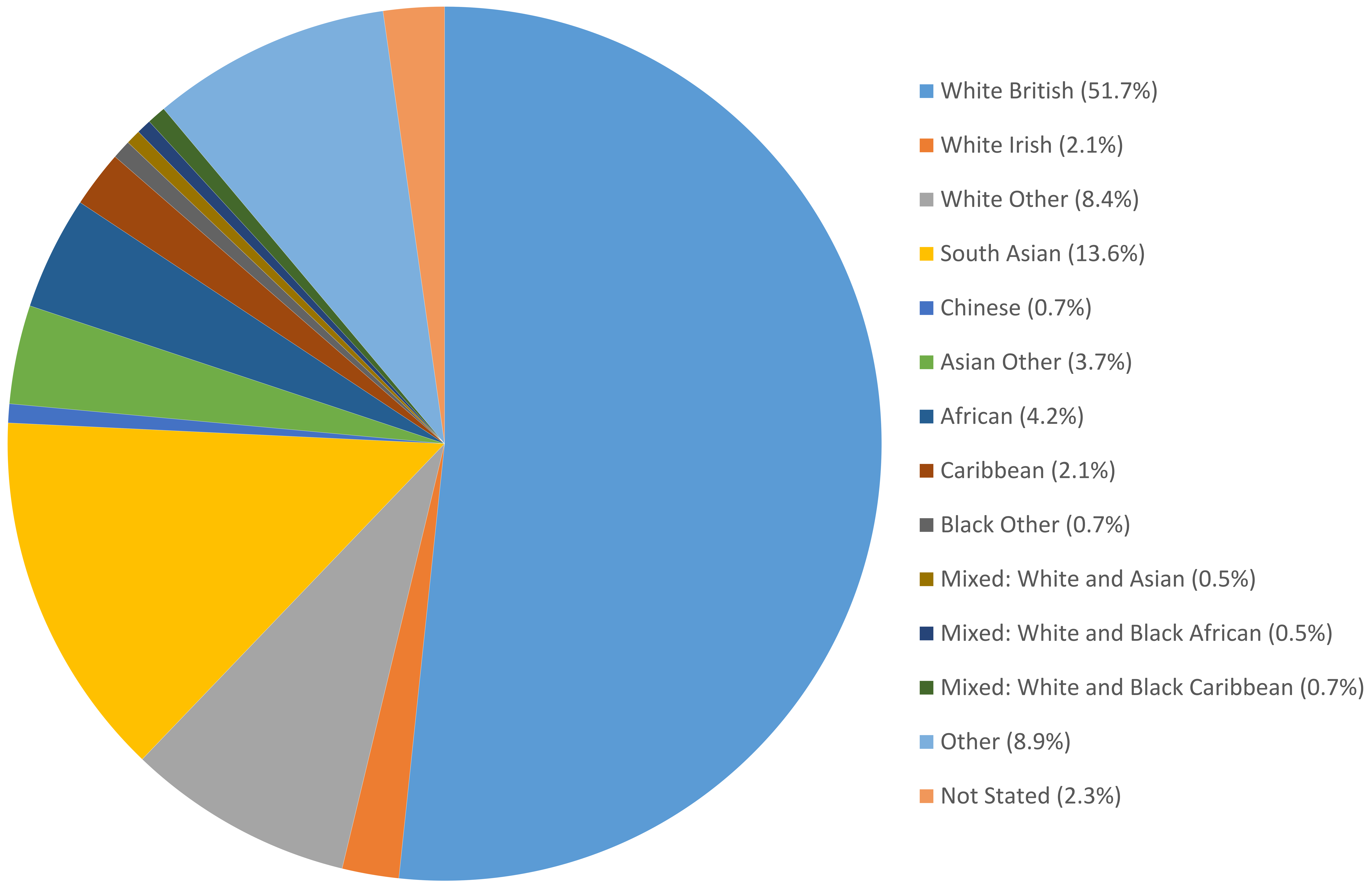
White British (51.7%)
White Irish (2.1%)
White Other (8.4%)
South Asian (13.6%)
Chinese (0.7%)
Asian Other (3.7%)
African (4.2%)
Caribbean (2.1%)
Black Other (0.7%)
Mixed: White and Asian (0.5%)
Mixed: White and Black African (0.5%)
Mixed: White and Black Caribbean (0.7%)
Other (8.9%)
Not Stated (2.3%)

Supplementary Figure 2. Ethnic distribution of probands. Pie chart shows proportions of each ethnicity for all 1287 IRD probands recruited from the genetics service to the “100,000 Genomes” project. The category “South Asian” includes those from Indian, Pakistani and Bangladeshi backgrounds.
