# Supplementary material for: Genetic Basis of Inherited Retinal Disease in a Molecularly Characterized Cohort of More Than 3000 Families from the United Kingdom
Source: Ophthalmology. 2020 Oct;127(10):1384–94. doi: 10.1016/j.ophtha.2020.04.008 (PMC7520514; doi:10.1016/j.ophtha.2020.04.008)
Supplement: Table S1 [file mmc3.pdf]

Supplementary Tables

Supplementary Table 1. Results (genes, numbers of families, numbers of affected individuals, published modes of inheritance) for the full cohort. LHON mutations have been combined.

	Chromo-somal location	Families affected (number)	Families affected (%)	Individuals affected (number)	Individuals affected (%)	Number of affected females	Number of affected males	Possible modes of inheritance
ABCA4	1p22.1	666	20.85	789	18.63	404	385	Recessive
USH2A	1q41	292	9.14	342	8.07	153	189	Recessive
RPGR	Xp11.4	164	5.13	263	6.21	34	229	X-linked
PRPH2	6p21.1	148	4.63	220	5.19	121	99	Dominant and Recessive
BEST1	11q12.3	125	3.91	168	3.97	68	100	Dominant and Recessive
RS1	Xp22.13	111	3.47	134	3.16	0	134	X-linked
RP1	8q12.1	106	3.32	170	4.01	88	82	Dominant and Recessive
RHO	3q22.1	105	3.29	177	4.18	98	79	Dominant and Recessive
CHM	Xq21.2	86	2.69	112	2.64	10	102	X-linked
CRB1	1q31.3	68	2.13	86	2.03	36	50	Recessive
PRPF31	19q13.42	57	1.78	94	2.22	56	38	Dominant
MYO7A	11q13.5	53	1.66	58	1.37	25	33	Recessive
OPA1	3q29	50	1.56	84	1.98	42	42	Dominant
CNGB3	8q21.3	44	1.38	55	1.30	28	27	Recessive
RPE65	1p31.2	39	1.22	51	1.20	21	30	Recessive and Dominant
EYS	6q12	38	1.19	43	1.02	20	23	Recessive
GUCY2D	17p13.1	37	1.16	54	1.27	23	31	Recessive and Dominant
PROM1	4p15.32	37	1.16	53	1.25	27	26	Recessive and Dominant
CNGA3	2q11.2	36	1.13	50	1.18	26	24	Recessive
RDH12	14q24.1	35	1.10	44	1.04	23	21	Recessive and Dominant
BBS1	11q13.5	32	1.00	37	0.87	15	22	Recessive
CACNA1F	Xp11.23	32	1.00	39	0.92	0	39	X-linked
RP2	Xp11.23	30	0.94	50	1.18	4	46	X-linked
TIMP3	22q12.3	29	0.91	64	1.51	38	26	Dominant
EFEMP1	2p16.1	26	0.81	39	0.92	28	11	Dominant
CEP290	12q21.32	25	0.78	35	0.83	14	21	Recessive
NR2E3	15q23	24	0.75	31	0.73	19	12	Recessive and Dominant
CRX	19q13.32	23	0.72	37	0.87	20	17	Dominant and Recessive
KCNV2	9p24.2	21	0.66	25	0.59	11	14	Recessive
PDE6B	4p16.3	20	0.63	22	0.52	13	9	Recessive and Dominant
CNGB1	16q21	19	0.59	20	0.47	12	8	Recessive
CYP4V2	4q35.2	19	0.59	27	0.64	14	13	Recessive
NMNAT1	1p36.22	19	0.59	21	0.50	11	10	Recessive
CDH23	10q22.1	18	0.56	20	0.47	12	8	Recessive
CERKL	2q31.3	18	0.56	20	0.47	9	11	Recessive
MERTK	2q13	17	0.53	18	0.42	10	8	Recessive
RP1L1	8p23.1	16	0.50	17	0.40	8	9	Dominant and Recessive
MTTL1	Mit.	16	0.50	17	0.40	13	4	Mitochondrial
ADGRV1	5q14.3	16	0.50	17	0.40	7	10	Recessive and Dominant
PRPF8	17p13.3	15	0.47	35	0.83	21	14	Dominant
USH1C	11p15.1	15	0.47	17	0.40	9	8	Recessive
LHON	Mit.	14	0.44	15	0.35	4	11	Mitochondrial
AIPL1	17q13.2	13	0.41	13	0.31	5	8	Recessive and Dominant
CDHR1	10q23.1	12	0.38	15	0.35	7	8	Recessive

ABCC6	16p13.11	12	0.38	12	0.28	7	5	Recessive and Dominant
KIF11	10q23.33	11	0.34	12	0.28	2	10	Dominant
IMPG2	3q12.3	11	0.34	13	0.31	4	9	Recessive
IQCB1	3q13.33	11	0.34	13	0.31	7	6	Recessive
LRP5	11q13.2	10	0.31	13	0.31	7	6	Dominant and Recessive
LCA5	6q14.1	10	0.31	10	0.24	8	2	Recessive
PDE6A	5q33.1	10	0.31	11	0.26	8	3	Recessive
PDE6C	10q23.33	10	0.31	11	0.26	9	2	Recessive
RDH5	14q24.1	10	0.31	10	0.24	3	7	Recessive
TTLL5	14q24.3	10	0.31	11	0.26	1	10	Recessive
IMPDH1	7q32.1	9	0.28	13	0.31	8	5	Dominant
C2ORF71	2p23.2	9	0.28	11	0.26	2	9	Recessive
MFSD8	4q28.2	9	0.28	11	0.26	5	6	Recessive
C1QTNF	11q23.3	8	0.25	12	0.28	6	6	Dominant
GUCA1A	6p21.1	8	0.25	13	0.31	6	7	Dominant
SNRNP200	2q11.2	8	0.25	11	0.26	6	5	Dominant
CLN3	16p11.2	8	0.25	8	0.19	4	4	Recessive
IFT140	16p13.3	8	0.25	10	0.24	3	7	Recessive
PCDH15	10q21.1	8	0.25	10	0.24	7	3	Recessive
RPGRIP1	14q11.2	8	0.25	9	0.21	5	4	Recessive
TULP1	6p21.31	8	0.25	9	0.21	5	4	Recessive
NYX	Xp11.4	8	0.25	8	0.19	0	8	X-linked
CDH3	16q22.1	7	0.22	7	0.17	1	6	Recessive
CLRN1	3q25.1	7	0.22	8	0.19	1	7	Recessive
FAM161A	2p15	7	0.22	9	0.21	3	6	Recessive
TRPM1	15q13.3	7	0.22	8	0.19	4	4	Recessive
WFS1	4p16.1	7	0.22	12	0.28	6	6	Recessive and Dominant
PRPF3	1q21.2	6	0.19	14	0.33	7	7	Dominant
AHI1	6q23.3	6	0.19	6	0.14	4	2	Recessive
C21ORF2	21q22.3	6	0.19	9	0.21	5	4	Recessive
FZD4	11q14.2	5	0.16	5	0.12	3	2	Dominant
KLHL7	7p15.3	5	0.16	7	0.17	3	4	Dominant
RP9	7p14.3	5	0.16	32	0.76	9	23	Dominant
BBS10	12q21.2	5	0.16	5	0.12	4	1	Recessive
DRAM2	1p13.3	5	0.16	5	0.12	3	2	Recessive
HGSNAT	8p11.21-p11.1	5	0.16	6	0.14	4	2	Recessive
MFRP	11q23.3	5	0.16	8	0.19	7	1	Recessive
PNPLA6	19p13.2	5	0.16	5	0.12	0	5	Recessive
RLBP1	15q26.1	5	0.16	9	0.21	6	3	Recessive
SPATA7	14q31.3	5	0.16	7	0.17	0	7	Recessive
NDP	Xp11.3	5	0.16	5	0.12	0	5	X-linked
COL11A1	1p21.1	4	0.13	4	0.09	4	0	Dominant
TOPORS	9q21.1	4	0.13	6	0.14	4	2	Dominant
BBS2	16q13	4	0.13	4	0.09	2	2	Recessive
GRM6	5q35.3	4	0.13	5	0.12	3	2	Recessive
OAT	10q26.13	4	0.13	4	0.09	4	0	Recessive
RBP3	10q11.22	4	0.13	6	0.14	2	4	Recessive
COL2A1	12q13.11	3	0.09	4	0.09	2	2	Dominant
NRL	14q11.2	3	0.09	17	0.40	9	8	Dominant and Recessive
ABHD12	20p11.21	3	0.09	3	0.07	0	3	Recessive

ALMS1	2p13.1	3	0.09	3	0.07	0	3	Recessive
ARHGEF18	19p13.3	3	0.09	3	0.07	2	1	Recessive
ARL6	3q11.2	3	0.09	3	0.07	1	2	Recessive
CABP4	11q13.1	3	0.09	4	0.09	2	2	Recessive
CNGA1	4p12	3	0.09	3	0.07	0	3	Recessive
LRAT	4q32.1	3	0.09	3	0.07	2	1	Recessive
ATXN7	3p14.1	2	0.06	2	0.05	0	2	Dominant
PAX2	10q24.31	2	0.06	3	0.07	1	2	Dominant
RIMS1	6q13	2	0.06	5	0.12	4	1	Dominant
ELOVL4	6q14.1	2	0.06	2	0.05	0	2	Dominant and Recessive
KCNJ13	2q37.1	2	0.06	3	0.07	0	3	Dominant and Recessive
AGBL5	2p23.3	2	0.06	3	0.07	3	0	Recessive
ATF6	1q23.3	2	0.06	2	0.05	2	0	Recessive
GPR179	17q12	2	0.06	2	0.05	1	1	Recessive
REEP6	19p13.3	2	0.06	2	0.05	1	1	Recessive
USH1G	17q25.1	2	0.06	2	0.05	1	1	Recessive
SAG	2q37.1	2	0.06	2	0.05	1	1	Recessive and Dominant
JAG1	20p12.2	1	0.03	1	0.02	1	0	Dominant
RP17	17q23.2	1	0.03	1	0.02	1	0	Dominant
TSPAN12	7q31.31	1	0.03	1	0.02	1	0	Dominant
IMPG1	6q14.1	1	0.03	1	0.02	0	1	Dominant and Recessive
MTTS2	Mit.	1	0.03	1	0.02	1	0	Mitochondrial
ADAM9	8q11.23	1	0.03	1	0.02	0	1	Recessive
ADAMTS18	16q23.1	1	0.03	2	0.05	1	1	Recessive
BBS12	4q27	1	0.03	1	0.02	0	1	Recessive
BBS4	15q24.1	1	0.03	1	0.02	0	1	Recessive
BBS5	2q31.1	1	0.03	1	0.02	1	0	Recessive
CACNA2D4	12p13.33	1	0.03	1	0.02	1	0	Recessive
FLVCR1	1q32.3	1	0.03	1	0.02	1	0	Recessive
GNAT2	1p13.3	1	0.03	1	0.02	0	1	Recessive
NPHP4	1p36.31	1	0.03	1	0.02	0	1	Recessive
PDE6G	17q25.3	1	0.03	2	0.05	1	1	Recessive
PEX1	7p21.2	1	0.03	1	0.02	1	0	Recessive
PHYH	10q13	1	0.03	1	0.02	0	1	Recessive
RBP4	10q23.33	1	0.03	1	0.02	1	0	Recessive
RGS9	17q24.1	1	0.03	1	0.02	0	1	Recessive
RGS9BP	19q13.12	1	0.03	2	0.05	2	0	Recessive
MKKS	20p12.2	1	0.03	1	0.02	0	1	Recessive
WDR19	4p14	1	0.03	1	0.02	0	1	Recessive
RGR	10q23.1	1	0.03	4	0.09	3	1	Recessive and Dominant
OPN1LW	Xq28	1	0.03	1	0.02	0	1	X-linked
