# Supplementary material for: Genetic Basis of Inherited Retinal Disease in a Molecularly Characterized Cohort of More Than 3000 Families from the United Kingdom
Source: Ophthalmology. 2020 Oct;127(10):1384–94. doi: 10.1016/j.ophtha.2020.04.008 (PMC7520514; doi:10.1016/j.ophtha.2020.04.008)
Supplement: Table S2 [file mmc4.pdf]

Supplementary Tables

Supplementary Table 2. Results (genes, numbers of families, numbers of affected individuals, published modes of inheritance) for individuals under 18 years.

Gene	Families affected (number)	Families affected (%)	Individuals affected (number)	Individuals affected (%)	Number of affected females	Number of affected males	Possible modes of inheritance
ABCA4	60	14.60	65	14.38	29	36	Recessive
RS1	29	7.06	33	7.30	0	33	X-linked
BEST1	20	4.87	24	5.31	7	17	Dominant and Recessive
CACNA1F	20	4.87	22	4.87	0	22	X-linked
RPGR	17	4.14	23	5.09	2	21	X-linked
CNGA3	16	3.89	18	3.98	6	12	Recessive
RPE65	16	3.89	17	3.76	10	7	Recessive and Dominant
CNGB3	14	3.41	17	3.76	6	11	Recessive
CRB1	13	3.16	14	3.10	5	9	Recessive
GUCY2D	13	3.16	13	2.88	8	5	Recessive and Dominant
OPA1	12	2.92	12	2.65	3	9	Dominant
NMNAT1	12	2.92	13	2.88	7	6	Recessive
CEP290	10	2.43	12	2.65	4	8	Recessive
KIF11	8	1.95	8	1.77	1	7	Dominant
RHO	7	1.70	7	1.55	3	4	Dominant and Recessive
AIPL1	7	1.70	7	1.55	3	4	Recessive and Dominant
RDH12	7	1.70	7	1.55	3	4	Recessive and Dominant
CHM	7	1.70	10	2.21	0	10	X-linked
PRPF31	6	1.46	5	1.11	3	2	Dominant
LCA5	6	1.46	6	1.33	5	1	Recessive
LRP5	5	1.22	6	1.33	3	3	Dominant and Recessive
CDH23	5	1.22	6	1.33	3	3	Recessive
NR2E3	5	1.22	6	1.33	3	3	Recessive
IQCB1	5	1.22	5	1.11	2	3	Recessive
PROM1	5	1.22	6	1.33	2	4	Recessive and Dominant
RP2	5	1.22	6	1.33	0	6	X-linked
FZD4	4	0.97	4	0.88	2	2	Dominant
CRX	4	0.97	5	1.11	5	0	Dominant and Recessive
TRPM1	4	0.97	5	1.11	3	2	Recessive
CLN3	4	0.97	4	0.88	1	3	Recessive
KCNV2	4	0.97	4	0.88	2	2	Recessive
MYO7A	4	0.97	4	0.88	1	3	Recessive
PNPLA6	4	0.97	4	0.88	1	3	Recessive
RPGRIP1	4	0.97	4	0.88	4	0	Recessive
NYX	4	0.97	4	0.88	0	4	X-linked
MERTK	3	0.73	3	0.66	2	1	Recessive
USH2A	3	0.73	3	0.66	0	3	Recessive
WFS1	3	0.73	3	0.66	3	0	Recessive and Dominant
NDP	3	0.73	3	0.66	0	3	X-linked
LHON	2	0.49	2	0.44	0	2	Mitochondrial inheritance
CABP4	2	0.49	2	0.44	0	2	Recessive
GPR179	2	0.49	2	0.44	1	1	Recessive
GRM6	2	0.49	2	0.44	1	1	Recessive

SPATA7	2	0.49	2	0.44	0	2	Recessive
PDE6B	2	0.49	2	0.44	2	0	Recessive and Dominant
COL2A1	1	0.24	1	0.22	1	0	Dominant
PRPF3	1	0.24	1	0.22	0	1	Dominant
IMPDH1	1	0.24	1	0.22	0	1	Dominant
PRPF8	1	0.24	1	0.22	1	0	Dominant
RIMS1	1	0.24	1	0.22	1	0	Dominant
TOPORS	1	0.24	1	0.22	1	0	Dominant
RP1	1	0.24	2	0.44	1	1	Dominant and Recessive
PRPH2	1	0.24	1	0.22	1	0	Dominant and Recessive
ADAM9	1	0.24	1	0.22	0	1	Recessive
AHI1	1	0.24	1	0.22	1	0	Recessive
ALMS1	1	0.24	1	0.22	0	1	Recessive
BBS10	1	0.24	1	0.22	0	1	Recessive
BBS2	1	0.24	1	0.22	0	1	Recessive
LRAT	1	0.24	1	0.22	0	1	Recessive
PDE6C	1	0.24	1	0.22	1	0	Recessive
PEX1	1	0.24	1	0.22	1	0	Recessive
RBP3	1	0.24	1	0.22	0	1	Recessive
RLBP1	1	0.24	1	0.22	1	0	Recessive
TULP1	1	0.24	1	0.22	1	0	Recessive
WDR19	1	0.24	1	0.22	0	1	Recessive
SAG	1	0.24	1	0.22	1	0	Recessive and Dominant
