# Supplementary material for: Genetic Basis of Inherited Retinal Disease in a Molecularly Characterized Cohort of More Than 3000 Families from the United Kingdom
Source: Ophthalmology. 2020 Oct;127(10):1384–94. doi: 10.1016/j.ophtha.2020.04.008 (PMC7520514; doi:10.1016/j.ophtha.2020.04.008)
Supplement: Table S3 [file mmc5.pdf]

Supplementary Tables

Supplementary Table 3. Genes in adult, but not pediatric cohort.

**Genes in adult but not pediatric cohort**
ABCC6
ABHD12
ADAMTS18
ADGRV1
AGBL5
ARHGEF18
ARL6
ATF6
ATXN7
BBS1
BBS12
BBS4
BBS5
C1QTNF
C21ORF2
C2ORF71
CACNA2D4
CDH3
CDHR1
CERKL
CLRN1
CNGA1
CNGB1
COL11A1
CYP4V2
DRAM2
EFEMP1
ELOVL4
EYS
FAM161A
FLVCR1
GNAT2
GUCA1A
HGSNAT
IFT140
IMPG1
IMPG2
JAG1
KCNJ13
KLHL7
MFRP
MFSD8
MKKS
MTTL1
MTTS2
NR2E3
NRL

OAT
OPN1LW
PAX2
PCDH15
PDE6A
PDE6G
PHYH
RBP4
RDH5
REEP6
RGR
RGS9
RGS9BP
RP17
RP1L1
RP9
SNRNP200
TIMP3
TSPAN12
TTLL5
USH1C
USH1G
