# Supplementary material for: Genetic Basis of Inherited Retinal Disease in a Molecularly Characterized Cohort of More Than 3000 Families from the United Kingdom
Source: Ophthalmology. 2020 Oct;127(10):1384–94. doi: 10.1016/j.ophtha.2020.04.008 (PMC7520514; doi:10.1016/j.ophtha.2020.04.008)
Supplement: Table S4 [file mmc6.pdf]

**Supplementary Tables**

Supplementary Table 4. Results (genes, numbers of families, numbers of affected individuals, published modes of inheritance) for the "current" cohort.

| Gene | Chromosomal location | Families affected (number) | Families affected (%) | Individuals affected (number) | Individuals affected (%) | Number of affected females | Number of affected males | Possible modes of inheritance |
|---|---|---|---|---|---|---|---|---|
| *ABCA4* | 1p22.1 | 583 | 22.30 | 656 | 20.96 | 348 | 308 | Recessive |
| *USH2A* | 1q41 | 230 | 8.80 | 261 | 8.34 | 118 | 143 | Recessive |
| *RPGR* | Xp11.4 | 131 | 5.01 | 181 | 5.78 | 17 | 164 | X-linked |
| *PRPH2* | 6p21.1 | 112 | 4.28 | 136 | 4.35 | 73 | 63 | Dominant and Recessive |
| *BEST1* | 11q12.3 | 103 | 3.94 | 123 | 3.93 | 52 | 71 | Dominant and Recessive |
| *RS1* | Xp22.13 | 83 | 3.18 | 96 | 3.07 | 0 | 96 | X-linked |
| *RP1* | 8q12.1 | 80 | 3.06 | 105 | 3.35 | 53 | 52 | Dominant and Recessive |
| *CHM* | Xq21.2 | 75 | 2.87 | 92 | 2.94 | 5 | 87 | X-linked |
| *RHO* | 3q22.1 | 74 | 2.83 | 99 | 3.16 | 55 | 44 | Dominant and Recessive |
| *CRB1* | 1q31.3 | 51 | 1.95 | 66 | 2.11 | 23 | 43 | Recessive |
| *PRPF31* | 19q13.42 | 48 | 1.84 | 54 | 1.73 | 32 | 22 | Dominant |
| *OPA1* | 3q29 | 44 | 1.68 | 65 | 2.08 | 28 | 37 | Dominant |
| *MYO7A* | 11q13.5 | 41 | 1.57 | 44 | 1.41 | 19 | 25 | Recessive |
| *CNGB3* | 8q21.3 | 40 | 1.53 | 49 | 1.57 | 26 | 23 | Recessive |
| *EYS* | 6q12 | 36 | 1.38 | 37 | 1.18 | 17 | 20 | Recessive |
| *PROM1* | 4p15.32 | 35 | 1.34 | 45 | 1.44 | 22 | 23 | Recessive and Dominant |
| *CNGA3* | 2q11.2 | 34 | 1.30 | 46 | 1.47 | 25 | 21 | Recessive |
| *RPE65* | 1p31.2 | 32 | 1.22 | 39 | 1.25 | 17 | 22 | Recessive and Dominant |
| *GUCY2D* | 17p13.1 | 28 | 1.07 | 35 | 1.12 | 17 | 18 | Recessive and Dominant |
| *RDH12* | 14q24.1 | 28 | 1.07 | 32 | 1.02 | 15 | 17 | Recessive and Dominant |
| *CACNA1F* | Xp11.23 | 28 | 1.07 | 32 | 1.02 | 0 | 32 | X-linked |
| *BBS1* | 11q13.5 | 26 | 0.99 | 27 | 0.86 | 10 | 17 | Recessive |
| *RP2* | Xp11.23 | 25 | 0.96 | 35 | 1.12 | 2 | 33 | X-linked |
| *EFEMP1* | 2p16.1 | 24 | 0.92 | 31 | 0.99 | 24 | 7 | Dominant |
| *CRX* | 19q13.32 | 20 | 0.77 | 26 | 0.83 | 12 | 14 | Dominant and Recessive |
| *NR2E3* | 15q23 | 19 | 0.73 | 24 | 0.77 | 15 | 9 | Recessive and Dominant |
| *PDE6B* | 4p16.3 | 19 | 0.73 | 19 | 0.61 | 10 | 9 | Recessive and Dominant |
| *TIMP3* | 22q12.3 | 18 | 0.69 | 32 | 1.02 | 20 | 12 | Dominant |
| *KCNV2* | 9p24.2 | 17 | 0.65 | 19 | 0.61 | 9 | 10 | Recessive |
| *CERKL* | 2q31.3 | 17 | 0.65 | 18 | 0.58 | 9 | 9 | Recessive |
| *CNGB1* | 16q21 | 17 | 0.65 | 18 | 0.58 | 11 | 7 | Recessive |
| *CDH23* | 10q22.1 | 16 | 0.61 | 18 | 0.58 | 11 | 7 | Recessive |
| *CEP290* | 12q21.32 | 15 | 0.57 | 22 | 0.70 | 9 | 13 | Recessive |
| *MERTK* | 2q13 | 15 | 0.57 | 16 | 0.51 | 8 | 8 | Recessive |
| *PRPF8* | 17p13.3 | 13 | 0.50 | 24 | 0.77 | 14 | 10 | Dominant |
| *CYP4V2* | 4q35.2 | 13 | 0.50 | 16 | 0.51 | 9 | 7 | Recessive |
| *RP1L1* | 8p23.1 | 12 | 0.46 | 12 | 0.38 | 6 | 6 | Dominant and Recessive |
| *LHON* | Mitochondrial | 12 | 0.46 | 13 | 0.42 | 3 | 10 | Mitochondrial inheritance |
| *MTTL1* | Mitochondrial | 11 | 0.42 | 11 | 0.35 | 8 | 3 | Mitochondrial inheritance |
| *IMPG2* | 3q12.3 | 11 | 0.42 | 11 | 0.35 | 4 | 7 | Recessive |
| *ADGRV1* | 5q14.3 | 11 | 0.42 | 12 | 0.38 | 5 | 7 | Recessive and Dominant |
| *CDHR1* | 10q23.1 | 10 | 0.38 | 12 | 0.38 | 5 | 7 | Recessive |
| *PDE6A* | 5q33.1 | 10 | 0.38 | 11 | 0.35 | 8 | 3 | Recessive |

| | | | | | | | | |
|---|---|---|---|---|---|---|---|---|
| *NMNAT1* | 1p36.22 | 10 | 0.38 | 10 | 0.32 | 5 | 5 | Recessive |
| *TTLL5* | 14q24.3 | 10 | 0.38 | 10 | 0.32 | 1 | 9 | Recessive |
| *IQCB1* | 3q13.33 | 9 | 0.34 | 10 | 0.32 | 6 | 4 | Recessive |
| *USH1C* | 11p15.1 | 9 | 0.34 | 10 | 0.32 | 6 | 4 | Recessive |
| *AIPL1* | 17q13.2 | 9 | 0.34 | 9 | 0.29 | 3 | 6 | Recessive and Dominant |
| *KIF11* | 10q23.33 | 8 | 0.31 | 9 | 0.29 | 2 | 7 | Dominant |
| *C2ORF71* | 2p23.2 | 8 | 0.31 | 9 | 0.29 | 2 | 7 | Recessive |
| *IFT140* | 16p13.3 | 8 | 0.31 | 9 | 0.29 | 3 | 6 | Recessive |
| *PDE6C* | 10q23.33 | 8 | 0.31 | 9 | 0.29 | 7 | 2 | Recessive |
| *ABCC6* | 16p13.11 | 8 | 0.31 | 8 | 0.26 | 3 | 5 | Recessive and Dominant |
| *IMPDH1* | 7q32.1 | 7 | 0.27 | 10 | 0.32 | 8 | 2 | Dominant |
| *GUCA1A* | 6p21.1 | 7 | 0.27 | 7 | 0.22 | 2 | 5 | Dominant |
| *LRP5* | 11q13.2 | 7 | 0.27 | 7 | 0.22 | 3 | 4 | Dominant and Recessive |
| *CLRN1* | 3q25.1 | 7 | 0.27 | 8 | 0.26 | 1 | 7 | Recessive |
| *MFSD8* | 4q28.2 | 7 | 0.27 | 8 | 0.26 | 5 | 3 | Recessive |
| *LCA5* | 6q14.1 | 7 | 0.27 | 7 | 0.22 | 5 | 2 | Recessive |
| *TULP1* | 6p21.31 | 7 | 0.27 | 7 | 0.22 | 4 | 3 | Recessive |
| *WFS1* | 4p16.1 | 7 | 0.27 | 11 | 0.35 | 6 | 5 | Recessive and Dominant |
| *PRPF3* | 1q21.2 | 6 | 0.23 | 10 | 0.32 | 5 | 5 | Dominant |
| *C1QTNF* | 11q23.3 | 6 | 0.23 | 9 | 0.29 | 5 | 4 | Dominant |
| *SNRNP200* | 2q11.2 | 6 | 0.23 | 8 | 0.26 | 4 | 4 | Dominant |
| *PCDH15* | 10q21.1 | 6 | 0.23 | 7 | 0.22 | 5 | 2 | Recessive |
| *RPGRIP1* | 14q11.2 | 6 | 0.23 | 7 | 0.22 | 4 | 3 | Recessive |
| *AHI1* | 6q23.3 | 6 | 0.23 | 6 | 0.19 | 4 | 2 | Recessive |
| *CLN3* | 16p11.2 | 6 | 0.23 | 6 | 0.19 | 4 | 2 | Recessive |
| *NYX* | Xp11.4 | 6 | 0.23 | 6 | 0.19 | 0 | 6 | X-linked |
| *C21ORF2* | 21q22.3 | 5 | 0.19 | 6 | 0.19 | 2 | 4 | Recessive |
| *FAM161A* | 2p15 | 5 | 0.19 | 6 | 0.19 | 2 | 4 | Recessive |
| *HGSNAT* | 8p11.21-p11.1 | 5 | 0.19 | 6 | 0.19 | 4 | 2 | Recessive |
| *BBS10* | 12q21.2 | 5 | 0.19 | 5 | 0.16 | 4 | 1 | Recessive |
| *CDH3* | 16q22.1 | 5 | 0.19 | 5 | 0.16 | 1 | 4 | Recessive |
| *DRAM2* | 1p13.3 | 5 | 0.19 | 5 | 0.16 | 3 | 2 | Recessive |
| *RDH5* | 14q24.1 | 5 | 0.19 | 5 | 0.16 | 1 | 4 | Recessive |
| *NDP* | Xp11.3 | 5 | 0.19 | 5 | 0.16 | 0 | 5 | X-linked |
| *RP9* | 7p14.3 | 4 | 0.15 | 15 | 0.48 | 5 | 10 | Dominant |
| *KLHL7* | 7p15.3 | 4 | 0.15 | 5 | 0.16 | 2 | 3 | Dominant |
| *TOPORS* | 9q21.1 | 4 | 0.15 | 5 | 0.16 | 4 | 1 | Dominant |
| *COL11A1* | 1p21.1 | 4 | 0.15 | 4 | 0.13 | 4 | 0 | Dominant |
| *FZD4* | 11q14.2 | 4 | 0.15 | 4 | 0.13 | 2 | 2 | Dominant |
| *RBP3* | 10q11.22 | 4 | 0.15 | 6 | 0.19 | 2 | 4 | Recessive |
| *RLBP1* | 15q26.1 | 4 | 0.15 | 5 | 0.16 | 4 | 1 | Recessive |
| *TRPM1* | 15q13.3 | 4 | 0.15 | 5 | 0.16 | 3 | 2 | Recessive |
| *MFRP* | 11q23.3 | 4 | 0.15 | 4 | 0.13 | 3 | 1 | Recessive |
| *OAT* | 10q26.13 | 4 | 0.15 | 4 | 0.13 | 4 | 0 | Recessive |
| *PNPLA6* | 19p13.2 | 4 | 0.15 | 4 | 0.13 | 1 | 3 | Recessive |
| *COL2A1* | 12q13.11 | 3 | 0.11 | 4 | 0.13 | 2 | 2 | Dominant |
| *NRL* | 14q11.2 | 3 | 0.11 | 10 | 0.32 | 5 | 5 | Dominant and Recessive |
| *CABP4* | 11q13.1 | 3 | 0.11 | 4 | 0.13 | 2 | 2 | Recessive |
| *GRM6* | 5q35.3 | 3 | 0.11 | 4 | 0.13 | 2 | 2 | Recessive |
| *ABHD12* | 20p11.21 | 3 | 0.11 | 3 | 0.10 | 0 | 3 | Recessive |

| | | | | | | | | |
|---|---|---|---|---|---|---|---|---|
| *ALMS1* | 2p13.1 | 3 | 0.11 | 3 | 0.10 | 0 | 3 | Recessive |
| *ARHGEF18* | 19p13.3 | 3 | 0.11 | 3 | 0.10 | 2 | 1 | Recessive |
| *ARL6* | 3q11.2 | 3 | 0.11 | 3 | 0.10 | 1 | 2 | Recessive |
| *CNGA1* | 4p12 | 3 | 0.11 | 3 | 0.10 | 0 | 3 | Recessive |
| *ATXN7* | 3p14.1 | 2 | 0.08 | 2 | 0.06 | 0 | 2 | Dominant |
| *PAX2* | 10q24.31 | 2 | 0.08 | 2 | 0.06 | 1 | 1 | Dominant |
| *KCNJ13* | 2q37.1 | 2 | 0.08 | 3 | 0.10 | 0 | 3 | Dominant and Recessive |
| *ATF6* | 1q23.3 | 2 | 0.08 | 2 | 0.06 | 2 | 0 | Recessive |
| *BBS2* | 16q13 | 2 | 0.08 | 2 | 0.06 | 2 | 0 | Recessive |
| *GPR179* | 17q12 | 2 | 0.08 | 2 | 0.06 | 1 | 1 | Recessive |
| *LRAT* | 4q32.1 | 2 | 0.08 | 2 | 0.06 | 1 | 1 | Recessive |
| *REEP6* | 19p13.3 | 2 | 0.08 | 2 | 0.06 | 1 | 1 | Recessive |
| *SPATA7* | 14q31.3 | 2 | 0.08 | 2 | 0.06 | 0 | 2 | Recessive |
| *USH1G* | 17q25.1 | 2 | 0.08 | 2 | 0.06 | 1 | 1 | Recessive |
| *SAG* | 2q37.1 | 2 | 0.08 | 2 | 0.06 | 1 | 1 | Recessive and Dominant |
| *RIMS1* | 6q13 | 1 | 0.04 | 4 | 0.13 | 3 | 1 | Dominant |
| *JAG1* | 20p12.2 | 1 | 0.04 | 1 | 0.03 | 1 | 0 | Dominant |
| *TSPAN12* | 7q31.31 | 1 | 0.04 | 1 | 0.03 | 1 | 0 | Dominant |
| *ELOVL4* | 6q14.1 | 1 | 0.04 | 1 | 0.03 | 0 | 1 | Dominant and Recessive |
| *IMPG1* | 6q14.1 | 1 | 0.04 | 1 | 0.03 | 0 | 1 | Dominant and Recessive |
| *MTTS2* | Mitochondrial | 1 | 0.04 | 1 | 0.03 | 1 | 0 | Mitochondrial inheritance |
| *ADAMTS18* | 16q23.1 | 1 | 0.04 | 2 | 0.06 | 1 | 1 | Recessive |
| *AGBL5* | 2p23.3 | 1 | 0.04 | 2 | 0.06 | 2 | 0 | Recessive |
| *RGS9BP* | 19q13.12 | 1 | 0.04 | 2 | 0.06 | 2 | 0 | Recessive |
| *ADAM9* | 8q11.23 | 1 | 0.04 | 1 | 0.03 | 0 | 1 | Recessive |
| *BBS12* | 4q27 | 1 | 0.04 | 1 | 0.03 | 0 | 1 | Recessive |
| *BBS5* | 2q31.1 | 1 | 0.04 | 1 | 0.03 | 1 | 0 | Recessive |
| *CACNA2D4* | 12p13.33 | 1 | 0.04 | 1 | 0.03 | 1 | 0 | Recessive |
| *FLVCR1* | 1q32.3 | 1 | 0.04 | 1 | 0.03 | 1 | 0 | Recessive |
| *GNAT2* | 1p13.3 | 1 | 0.04 | 1 | 0.03 | 0 | 1 | Recessive |
| *MKKS* | 20p12.2 | 1 | 0.04 | 1 | 0.03 | 0 | 1 | Recessive |
| *NPHP4* | 1p36.31 | 1 | 0.04 | 1 | 0.03 | 0 | 1 | Recessive |
| *PEX1* | 7p21.2 | 1 | 0.04 | 1 | 0.03 | 1 | 0 | Recessive |
| *PHYH* | 10q13 | 1 | 0.04 | 1 | 0.03 | 0 | 1 | Recessive |
| *RBP4* | 10q23.33 | 1 | 0.04 | 1 | 0.03 | 1 | 0 | Recessive |
| *WDR19* | 4p14 | 1 | 0.04 | 1 | 0.03 | 0 | 1 | Recessive |
| *RGR* | 10q23.1 | 1 | 0.04 | 1 | 0.03 | 1 | 0 | Recessive and Dominant |
| *OPN1LW* | Xq28 | 1 | 0.04 | 1 | 0.03 | 0 | 1 | X-linked |
